# Secondary Osteonecrosis of Both Tibial and Femoral Condyles Induced by Dexamethasone Overuse: A Case Report

**DOI:** 10.7759/cureus.84595

**Published:** 2025-05-22

**Authors:** Taha El Aissaoui, Ayman Ben Abdellah, Adnane Lachkar, Hicham Yacoubi, Najib Abdeljaouad

**Affiliations:** 1 Department of Traumatology and Orthopaedics, Mohammed VI University Hospital Centre, Oujda, MAR; 2 Faculty of Medicine and Pharmacy, Mohammed First University, Oujda, MAR

**Keywords:** dexamethasone, femur, knee, knee osteonecrosis, mri, tibia

## Abstract

Osteonecrosis of the knee is a rare but serious condition that can lead to progressive joint destruction and functional impairment. Secondary osteonecrosis, particularly related to corticosteroid overuse, represents a challenging and often under-recognized clinical entity. We report the case of a 32-year-old female from Eastern Morocco who developed severe bilateral knee osteonecrosis involving both the distal femur and proximal tibia following prolonged self-medication with high-dose dexamethasone. The patient presented with chronic knee pain, stiffness, and functional limitation. Imaging studies, including magnetic resonance imaging, revealed characteristic osteonecrotic lesions with a serpiginous border and the double line sign in both the femoral and tibial components. Laboratory investigations excluded infectious, inflammatory, and hematologic causes. Given the extensive osteonecrotic damage and the patient's refusal of surgical intervention, a conservative management strategy combining physical rehabilitation, lifestyle modification, protected weight-bearing, and analgesia was adopted. This case highlights the devastating potential of unsupervised corticosteroid use and emphasizes the importance of early recognition, comprehensive patient history, and timely imaging to prevent severe joint degeneration. To our knowledge, this is the first reported case in Eastern Morocco with such extensive topographic involvement secondary to dexamethasone overuse, contributing valuable insight to the literature on corticosteroid-induced musculoskeletal complications.

## Introduction

Since Ahlbäck first described knee osteonecrosis in 1968 [1], substantial progress has been made in understanding its clinical progression and underlying pathophysiology. This debilitating disease often advances to end-stage osteoarthritis, resulting in considerable functional impairment and a diminished quality of life. Although its overall incidence remains relatively low, the knee is recognized as the second most frequently affected joint after the hip [2]. Despite being an uncommon cause of knee pain, osteonecrosis presents ongoing diagnostic and therapeutic challenges. Treatment strategies are evolving, guided by an increasingly detailed understanding of the disease’s origins and mechanisms [1].

Secondary osteonecrosis of the knee (SOK) is a progressive bone disorder with a multifactorial etiology, accounting for approximately 10% of all osteonecrosis cases [3]. Systemic corticosteroid use is a well-established risk factor, particularly at high doses or over extended periods [4]. The pathological mechanisms implicated include disturbances in lipid metabolism, vascular endothelial injury, and direct cytotoxic effects on osteocytes and bone marrow cells [5].

In this report, we present a particularly uncommon and severe case of secondary knee osteonecrosis, distinguished by its extensive anatomical involvement and association with prolonged dexamethasone overuse. To our knowledge, this is the first such case reported from Morocco, further highlighting the need for heightened clinical awareness of corticosteroid-related osteonecrosis in both endemic and underreported regions.

## Case presentation

We present the case of a 32-year-old female patient with a medical history of hypothyroidism due to autoimmune thyroiditis, managed with levothyroxine at a dose of 25 mg per day.

**Figure 1:**
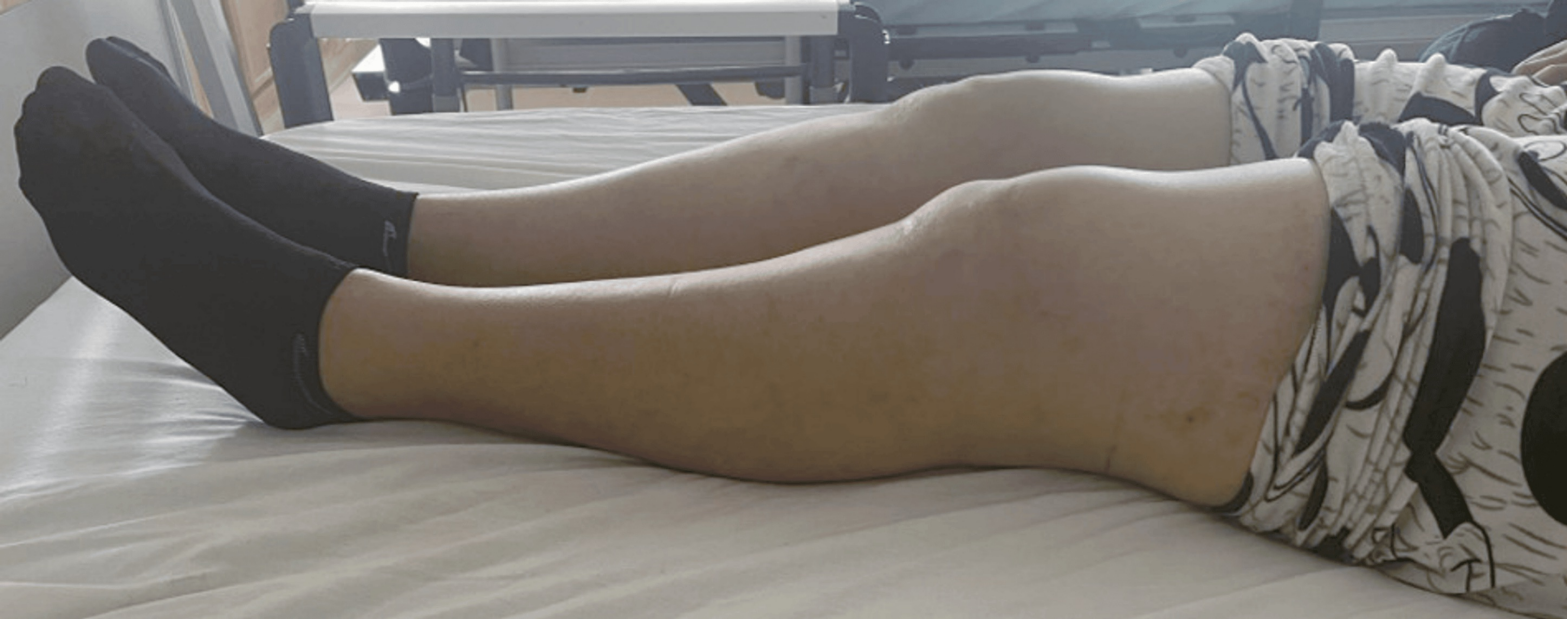
Clinical image showing full extension of the left knee.

**Figure 2:**
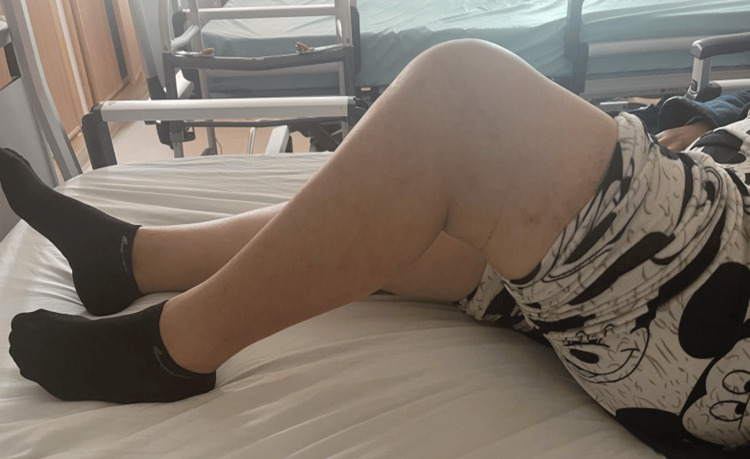
Clinical image showing limited flexion of the left knee.

Eight months prior to her first medical consultation, the patient experienced a fall impacting the left knee, which led to progressively worsening pain and stiffness. On physical examination, she exhibited a limping gait, quadriceps muscle atrophy, and tenderness on palpation of the femoral condyles. No visible deformity or swelling was noted. The range of motion was limited to 0°-0°-80° (Figures 1, 2).

**Figure 3:**
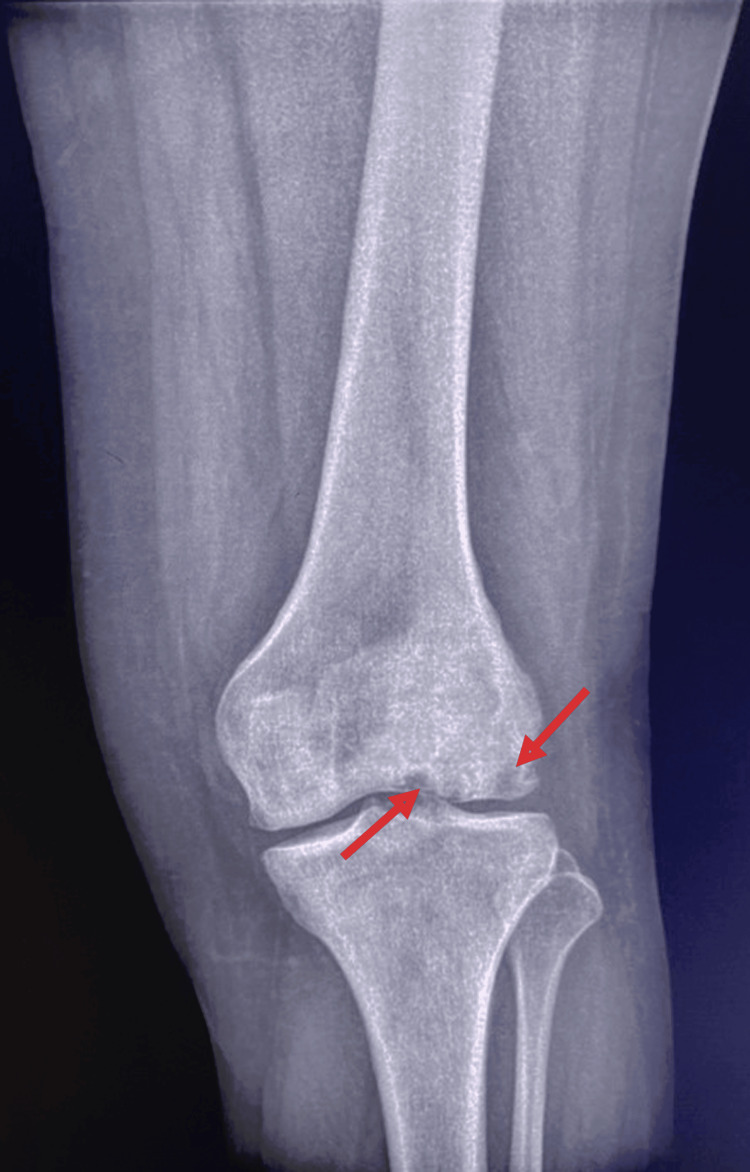
Radiograph of the left knee demonstrating osteolytic areas within the distal femur.

**Figure 4:**
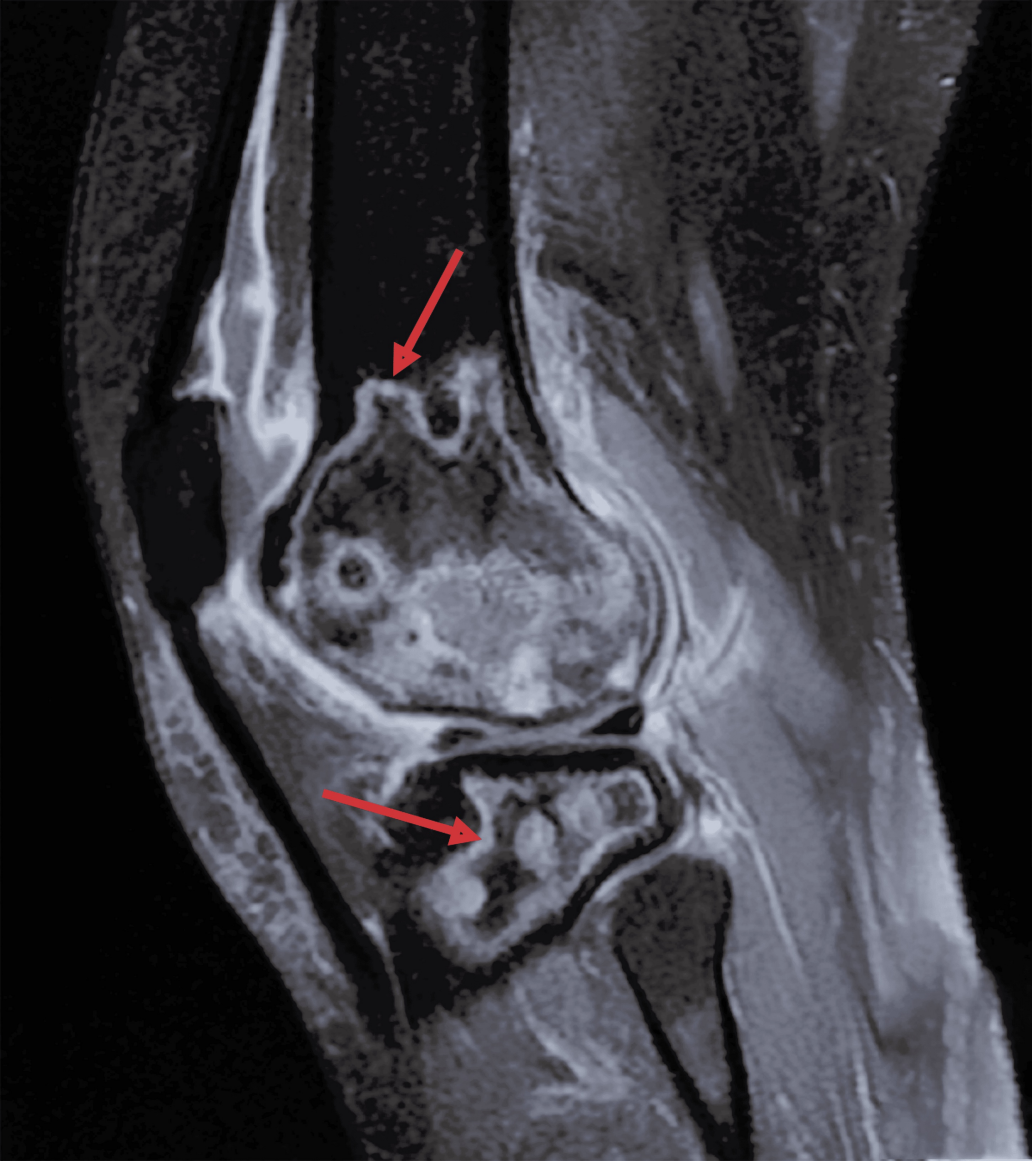
T2 fat-saturated sagittal MRI of the left knee showing osteonecrosis of both femur and tibia. Medullary meta-epiphyseal lesions in the proximal tibia and distal femur, exhibiting a serpiginous border and a double-line sign.

**Figure 5:**
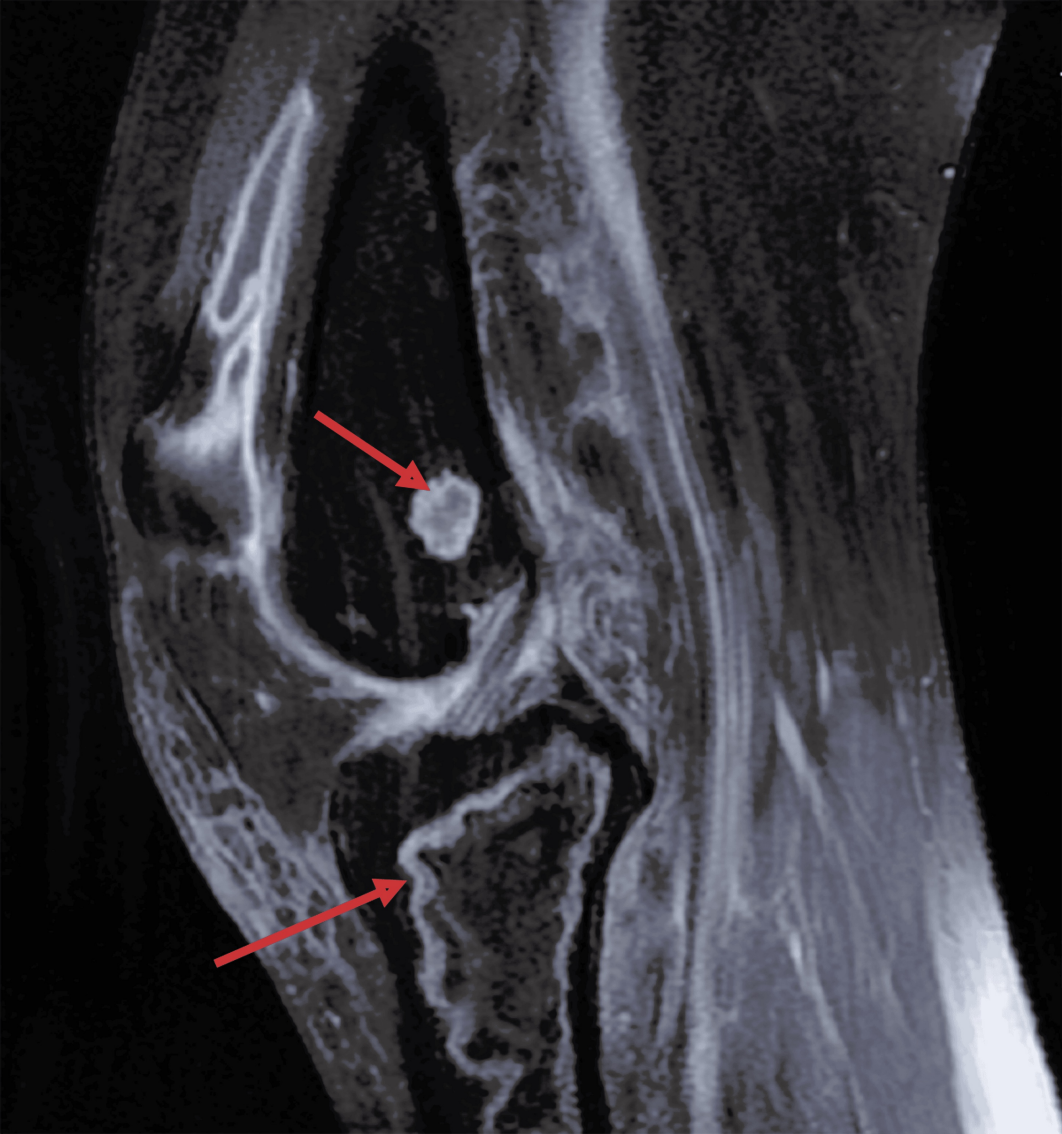
T2 fat-saturated sagittal MRI of the left knee showing osteonecrosis of both femur and tibia. Medullary meta-epiphyseal lesions in the proximal tibia and distal femur, exhibiting a serpiginous border and a double-line sign.

Standard radiographs of the left knee revealed osteolytic lesions in the distal aspect of the lateral femoral condyle (Figure 3). MRI confirmed the diagnosis of aseptic osteonecrosis involving both the distal femur and proximal tibia. Magnetic resonance imaging revealed the presence of medullary meta-epiphyseal lesions in the proximal tibia and distal femur with a serpiginous border and double line sign, and subchondral necrosis of femoral and tibial condyles was evident (Figures 4, 5).

In light of these clinical and radiological findings, laboratory investigations were conducted and returned within normal ranges or negative. Abdominal examination revealed no hepatomegaly or splenomegaly. Blood parameters were unremarkable, with normal hemoglobin, white blood cell count, erythrocyte sedimentation, C-reactive protein, liver function, and kidney function tests. Rheumatoid factor, anti-cyclic citrullinated antibody, and anti-nuclear antibody were negative. The sickling test was negative, and hemoglobin electrophoresis was normal. Upon further questioning, the patient disclosed a history of self-medicating with dexamethasone at a dose of 7.5 mg daily for 4 months, which she had initially failed to mention during her first visit. Given the severity of the osteonecrotic lesions and the patient's refusal to undergo surgical intervention, a conservative management strategy was adopted. This approach included a structured physiotherapy program aimed at enhancing muscle strength and restoring joint range of motion, along with modifications in daily activities to reduce mechanical stress on the affected knee. The patient was also advised to use a crutch on the contralateral side to offload weight from the affected limb and was prescribed appropriate analgesic therapy to manage pain.

Throughout 10 months of follow-up, the patient reported a reduction in pain intensity and demonstrated a modest improvement in the ability to perform daily activities, although functional recovery remained limited.

## Discussion

Secondary osteonecrosis of the knee (SOK) remains a rare and frequently under-recognized condition. Although the femoral head is the most common site of osteonecrosis, the knee is the second most commonly affected, particularly among younger patients, often displaying bilateral and multifocal lesions. The etiologies can be categorized as either direct - such as sickle cell disease, caisson disease, Gaucher’s disease, or myeloproliferative disorders - or indirect, including excessive alcohol consumption, corticosteroid use, smoking, and obesity [5]. Between 10% and 30% of secondary osteonecrosis cases are attributed to corticosteroid therapy [6]. Dexamethasone, a potent corticosteroid, has seen increased usage, especially during the COVID-19 pandemic, which has been associated with a corresponding rise in steroid-induced osteonecrosis [7]. The pathogenesis involves fat embolism, microvascular impairment, and osteocyte apoptosis, all contributing to compromised subchondral bone circulation [8].

Clinically, most patients with secondary knee osteonecrosis present with bilateral involvement in more than 80% of cases [5], typically reporting a gradual onset of localized pain. While the femoral condyle is the most frequently involved structure, osteonecrosis affecting the tibial condyle occurs in approximately 20% of cases [9]. Diagnostic imaging begins with anteroposterior and lateral radiographs, although magnetic resonance imaging (MRI) remains the most sensitive modality [5]. In early stages, MRI typically reveals areas of low signal intensity in the subchondral bone on T2-weighted images, often accompanied by focal epiphyseal depressions. The characteristic "double line sign," consisting of a low-signal outer rim and a high-signal inner rim on T2-weighted sequences, is considered diagnostic [10].

Management strategies vary depending on the stage and extent of the lesion, ranging from conservative approaches to surgical interventions. Non-operative management focuses on pain relief and delaying structural collapse through the use of non-steroidal anti-inflammatory drugs (NSAIDs), physical modalities such as extracorporeal shock wave therapy and ultrasound, and protected weight-bearing regimens. Surgical options include joint-preserving procedures and, in more advanced cases, total joint arthroplasty.

Simultaneous osteonecrosis involving both the distal femur and the proximal tibia remains exceedingly rare. Only a limited number of cases have been reported, including those described by Agarwala et al. [11] and Sondur et al. [12], where MRI confirmed the involvement of both femoral and tibial components. The case reported here represents the first documented instance of such an extensive steroid-induced osteonecrosis pattern in Eastern Morocco and remains among the few described globally.

This case underscores the severe consequences of unsupervised corticosteroid use and emphasizes the necessity of early diagnosis through vigilant clinical assessment and timely imaging. Additionally, it offers valuable contributions to the growing body of literature on the musculoskeletal complications associated with corticosteroid therapy, particularly in regions where access to medical oversight may be limited.

## Conclusions

This case underscores the potentially devastating consequences of unregulated corticosteroid use, particularly dexamethasone, which may lead to extensive and multifocal osteonecrosis of the knee. The simultaneous involvement of the distal femur and proximal tibia remains exceptionally rare. To our knowledge, this is the first reported case of such severity and distribution in Eastern Morocco. Early recognition, detailed patient history, including medication use, and prompt imaging are crucial for timely diagnosis and management. This report adds to the growing body of literature on steroid-induced osteonecrosis. It highlights the need for increased clinical vigilance, especially in regions where corticosteroids are accessible without strict medical oversight.
